# Supplementary figures and images for: Optimal management of peripancreatic fluid collection with postoperative pancreatic fistula after distal pancreatectomy: Significance of computed tomography values for predicting fluid infection
Source: PLoS One. 2021 Nov 9;16(11):e0259701. doi: 10.1371/journal.pone.0259701 (PMC8577730; doi:10.1371/journal.pone.0259701)

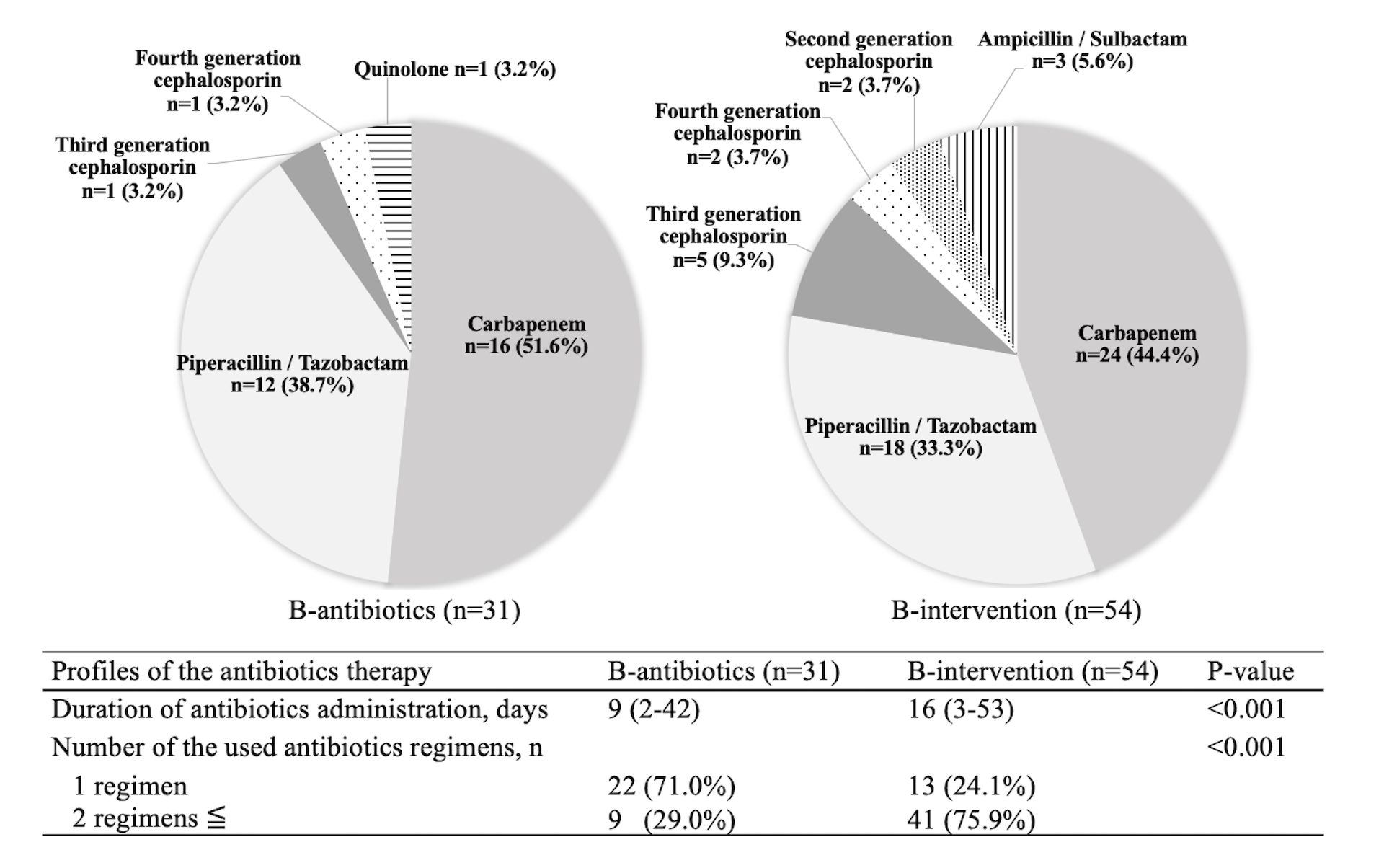

Supplement: S1 Fig — Carbapenem and piperacillin/tazobactam were used frequently in both groups. The duration of antibiotic therapy was significantly longer while the number of used antibiotics regimens was significantly greater in the B-intervention group than in the B-antibiotics group. (TIF) [file pone.0259701.s001.tif]
