# Supplementary material for: Optimal management of peripancreatic fluid collection with postoperative pancreatic fistula after distal pancreatectomy: Significance of computed tomography values for predicting fluid infection
Source: PLoS One. 2021 Nov 9;16(11):e0259701. doi: 10.1371/journal.pone.0259701 (PMC8577730; doi:10.1371/journal.pone.0259701)
Supplement: S1 Table — (DOCX) [file pone.0259701.s002.docx]

| **S1 Table. Comparison of patient backgrounds and POPF rates between the open and laparoscopic surgery groups.** | | | | |
| --- | --- | --- | --- | --- |
| Backgrounds and POPF rates | **Open (n = 171)** | **Laparocopy (n = 88)** | **P-value** |  |
| **Preoperative characteristics** |  |  |  |  |
| Sex, male/female | 106/65 | 45/43 | 0.093 |  |
| Age, years | 68 (3–89) | 64 (10–87) | 0.079 |  |
| BMI, kg/m^2^ | 21.2 (13.6–34.7) | 21.9 (15.4–30.5) | 0.063 |  |
| DM, n | 51 (29.8%) | 15 (17.1%) | **0.025** |  |
| PDAC, n | 96 (56.1%) | 11 (12.5%) | **<0.001** |  |
| Preoperative therapy, n | 64 (37.4%) | 4 (4.6%) | **<0.001** |  |
| PNI | 46.4 (30.9–62.1) | 50.5 (36.5–62.1) | **<0.001** |  |
| **Intraoperative characteristics** |  |  |  |  |
| Operation time, min | 338 (138–830) | 300 (132–754) | 0.056 |  |
| Blood loss, mL | 655 (0–11,300) | 100 (0–2,000) | **<0.001** |  |
| without splenectomy, n | 8 (4.7%) | 18 (20.5%) | **<0.001** |  |
| Simultaneous resection of alimentary tract, n | 27 (15.8%) | 0 (0%) | **<0.001** |  |
| **Incidence of POPF** |  |  | 0.599 |  |
| Non (%, non/BL), n | 111 (64.9%, 102/9) | 60 (68.2%, 39/21) |  |  |
| CR-POPF (%, grade B/C), n | 60 (35.1%, 58/2) | 28 (31.8%, 27/1) |  |  |

Data are expressed as number (percentage) or median (range).

POPF: postoperative pancreatic fistula, BMI: body mass index, DM: diabetes mellitus, PDAC: pancreatic ductal adenocarcinoma, PNI: prognostic nutritional index, CR: clinically relevant, BL: biochemical leak
