# Supplementary material for: Optimal management of peripancreatic fluid collection with postoperative pancreatic fistula after distal pancreatectomy: Significance of computed tomography values for predicting fluid infection
Source: PLoS One. 2021 Nov 9;16(11):e0259701. doi: 10.1371/journal.pone.0259701 (PMC8577730; doi:10.1371/journal.pone.0259701)
Supplement: S2 Table — (DOCX) [file pone.0259701.s003.docx]

| **S2 Table. Comparison of the treatments for grade B POPF between the open and laparoscopic surgery groups.** | | | | |
| --- | --- | --- | --- | --- |
| Managements of grade B POPF | **Open (n = 58)** | **Laparocopy (n = 27)** | **P-value** |  |
| **Postoperative blood examination** |  |  |  |  |
| White blood cell count on POD 3, /μL | 12,590 (5,870–23,220) | 13,720 (8,460–25,450) | 0.406 |  |
| CRP on POD3, mg/dl | 16.00 (5.15–36.71) | 17.59 (8.85–38.18) | 0.337 |  |
| White blood cell count on POD 6-7, /μL | 9,800 (4,990–14,900) | 9,610 (7,740–14,180) | 0.848 |  |
| CRP on POD 6-7, mg/dL | 5,97 (0.97-19,03) | 7.26 (1.97-21.25) | 0.289 |  |
| **First CT evaluation of PFCs** |  |  |  |  |
| POD | 6 (2–17) | 6 (2–17) | 0.299 |  |
| Incidence of PFCs, n | 48 (82.8%) | 17 (63.0%) | **0.045** |  |
| CT value, HU | 16.9 (6.9–33.7) | 18.1 (11.8–28.8) | 0.397 |  |
| CT volume, mL | 21.0 (0.0–274.5) | 24.5 (0–94.8) | 0.839 |  |
| **Managements of grade B POPF** |  |  | **<0.001** |  |
| B-antibiotics, n | 13 (22.4%) | 18 (66.7%) |  |  |
| B-intervention, n | 45 (77.6%) | 9 (33.3%) |  |  |
| persistent drainage only > 3 weeks, n | 4 (6.9%) | 3 (11.1%) |  |  |
| Additional drainage for PFCs, n | 37 (63.8%) | 6 (22.2%) |  |  |
| Non-infected/Infected, n | 26/11 | 4/2 | 0.648 |  |
| CT-guided, n | 30 | 4 |  |  |
| US-guided, n | 3 | 2 |  |  |
| EUS-guided, n | 4 | 0 |  |  |
| EPD, n | 9 | 0 |  |  |
| TAE, n | 5 | 1 |  |  |
| Endoscopic hemostasis, n | 1 | 1 |  |  |
| **Clinical outcome** |  |  |  |  |
| Fistula-related readmission, n | 4 (6.9%) | 2 (7.4%) | 0.391 |  |
| Length of hospital stay, days | 42 (7–248) | 17 (9–53) | **<0.001** |  |
| Overall complications CD ≧ 3a, n | 46 (79.3%) | 8 (29.6%) | **<0.001** |  |

Data are expressed as number (percentage) or median (range).

POPF: postoperative pancreatic fistula, POD: postoperative day, CRP: C-reactive protein, PFCs: peripancreatic fluid collections, CT: computed tomography, HU: hounsfield units, US: ultrasonography, EUS: endoscopic ultrasonography, EPD: endoscopic pancreatic drainage, TAE: transcatheter arterial embolization, CD: Clavien–Dindo classification
